# Spatial Distribution of the Pepper Blight (*Phytophthora capsici*) Suppressive Microbiome in the Rhizosphere

**DOI:** 10.3389/fpls.2021.748542

**Published:** 2022-01-21

**Authors:** Huixiu Li, Ning Wang, Jia Ding, Yingjie Liu, Xiaoyan Ding, Yuquan Wei, Ji Li, Guo-chun Ding

**Affiliations:** ^1^Beijing Key Laboratory of Biodiversity and Organic Farming, College of Resources and Environmental Science, China Agricultural University, Beijing, China; ^2^Tangshan Normal University, Tangshan, China; ^3^Organic Recycling Institute (Suzhou) of China Agricultural University, Suzhou, China

**Keywords:** spatial distribution, microbial community, rhizosphere, pepper blight, disease- suppressive soil

## Abstract

The properties of plant rhizosphere are dynamic and heterogeneous, serving as different habitat filters for or against certain microorganisms. Herein, we studied the spatial distribution of bacterial communities in the rhizosphere of pepper plants treated with a disease-suppressive or non-suppressive soil. The bacterial richness was significantly (*p* < 0.05) higher in plants treated with the disease-suppressive soil than in those treated with the non-suppressive soil. Bacterial richness and evenness greatly differed between root parts, with decrease from the upper taproot to the upper fibrous root, the lower taproot, and the lower fibrous root. As expected, the bacterial community in the rhizosphere differed between suppressive and non-suppressive soil. However, the spatial variation (36%) of the bacterial community in the rhizosphere was much greater than that explained by soils (10%). Taxa such as subgroups of Acidobacteria, *Nitrosospira*, and *Nitrospira* were known to be selectively enriched in the upper taproot. *In vitro Bacillus* antagonists against *Phytophthora capsici* were also preferentially colonized in the taproot, while the genera such as *Clostridium*, *Rhizobium*, *Azotobacter*, *Hydrogenophaga*, and *Magnetospirillum* were enriched in the lower taproot or fibrous root. In conclusion, the spatial distribution of bacterial taxa and antagonists in the rhizosphere of pepper sheds light on our understanding of microbial ecology in the rhizosphere.

## Introduction

The abundance and diversity of microorganisms that inhabit the rhizosphere of plants play key roles in maintaining plant nutrients and health (Berg and Smalla, 2009; Mendes et al., 2011, 2013; Faria et al., 2020; Yu et al., 2020). Rhizospheric microbiomes may increase crop nutrients acquisition and resistance to environmental stresses (Ahkami et al., 2017), thus decreasing the excessive use of chemical fertilizers or pesticides. Mechanisms governing the assembly of microbial communities in the rhizosphere are the foundation for building beneficial rhizospheric microbiomes (Bai et al., 2015; Heijden and Hartmann, 2016; Gu et al., 2020; Trivedi et al., 2020). Progress in high-throughput sequencing and its application in metagenomics have advanced our understanding of microbial ecology in the rhizosphere. Several biotic and abiotic factors, such as plant species and development (Bell et al., 2015; Ma et al., 2019), soil animals (Jiang et al., 2017), viruses (Pratama et al., 2020), inoculation of microbial consortia (Zhang et al., 2019), invasion of pathogens (Yin et al., 2021), land use (Wang C. et al., 2021), fertilization (Ding et al., 2020), and tillage (Li et al., 2021), may cause changes in the composition and functions of microbial communities inhabiting the rhizosphere. Shifts in the microbial community may increase the resistance of plants to diseases, such as the common scab of potato (Shi et al., 2019) and pepper blight (Li et al., 2019; Zhang et al., 2019). In addition, the interactions among rhizospheric microorganisms may also influence the recruitment of beneficial plant microorganisms (Tao et al., 2020).

So far, the microbiome in the rhizosphere of plants has been studied using the whole root system (Barillot et al., 2013). However, the properties of the rhizosphere are very likely dynamic and heterogeneous (Vetterlein et al., 2020). For example, root systems are continuously differentiating and changing during the growth of plants (Hodge, 2006; Gruber et al., 2013). In addition, at finer scales, the soils vary greatly with respect to their redox potential (Smith et al., 2021), pH (Blossfeld et al., 2011), and availability of different nutrients or enzyme activities (Koop-Jakobsen and Wenzhöfer, 2015; Kuzyakov and Razavi, 2019). These factors affect the structures of soil microbial communities (Nunan, 2017). Microbial niches in the rhizosphere may serve as different habitat filters for or against certain other microorganisms. Thus, responses of the microbiome in the rhizosphere to different factors might be a “sum up” of the changes in these microbial niches. The characteristics of microbial niches are likely to be shaped synergistically by plant root exudates, by the physicochemical properties surrounding the root, or by the feedbacks of the microorganisms. Root exudates initiate and mediate the activities of microorganisms and their interactions with plants (Berg and Smalla, 2009). Taproot and fibrous roots differ greatly in terms of the release of root exudates, and most root exudates are released from the fibrous roots or root hairs (Trivedi et al., 2020). Physicochemical and biological gradients along the soil profile also differ in common; therefore, the vertical surroundings of taproots or fibrous roots might differ greatly. Thus, we hypothesize that the composition of the bacterial community recruited in the rhizosphere would depend on the combination of root type and its vertical surroundings and that habitat filter is a major driver of rhizosphere communities at a finer scale.

In this study, the spatial distribution of bacterial communities recruited from the disease-suppressive or non-suppressive soil was explored in the rhizosphere of pepper plants. We focused on two main questions: (1) What is the spatial distribution of the bacterial community and the antagonists in the rhizosphere of peppers plants? (2) To which level such spatial distributions might be influenced by different soil microbiomes?

## Materials and Methods

### Long-Term Greenhouse and Bioassay Experiments

Soil samples were collected from a long-term greenhouse experiment conducted at the Quzhou Experimental Station (36° 52′ N, 115° 01′ E), Hebei, China. That experiment contains organic and conventional farming systems, which followed the same scheme of crop rotation, tillage, and irrigation. Details of the experiment have been described previously (Han et al., 2017; Ding et al., 2019). Plant diseases such as late blight and powdery mildew on tomato (*Solanum lycopersicum* L.) or cucumber (*Cucumis sativus* L.) are less severe in the organic farming system than in the conventional farming systems (Yang et al., 2009a,b). In the climate chamber experiment, an incidence of pepper blight was 41% lower in the soil from the organic farming system than that from the conventional farming system, possibly due to the enhanced *Bacillus* antagonists in the rhizosphere (Li et al., 2019). Long-term organic farming likely increased the suppressive power of the soil toward plant diseases; henceforth, the soils samples from the organic and conventional farming systems are referred to as disease-suppressive and non-suppressive soils, respectively.

For each system, 75 cores (2 cm) of soil from the top layer (1–20 cm) were collected, mixed thoroughly, and passed through 2-mm mesh and stored at 4°C. The bioassay was performed as follows: after surface sterilization, “Cayenne” pepper (*Capsicum annuum*) seeds (Zhong liang xin) were germinated at 30°C in the dark, and after germination, the seeds were set at the seedling point suggested on the PhytoTC seed germination pouch (18 cm × 12.5 cm) and soaked in a mixture of 10 g of disease-suppressive or non-suppressive soil and 20 mL sterilized Hoagland solution. The seedlings were grown for 28 days in a growth chamber (Hangzhou Lvbo Instrument Co., Ltd., LB-1000D-LED) at 30°C, 70% relative humidity, and under a 12-h light (15,000 lx) period. Fresh standard Hoagland solution (20 ml) was re-added in the pouch. Each treatment consisted of four replicates, and each replicates contained two plants. The root system adhering to the seed germination paper was dissected into four different parts (e.g., upper taproot, upper fibrous root, lower taproot, and lower fibrous root), and the roots were carefully taken from the pouch. Different parts of root were vortexed vigorously in a 0.85% NaCl solution for 5 min (King et al., 2021). The pellet for DNA extraction was collected by a centrifugation (Eppendorf 5804R) at 6,000*g* for 5 min. All these samples were stored at −20°C prior to bacterial isolation or DNA extraction.

### Total Community DNA Extraction and Amplification, Purification, and Sequencing of 16S *rRNA*

Total community DNA was extracted using the FastDNA Spin Kit from the soil samples according to the instruction of the manufacturer. Amplification of 16S *rRNA* fragments was performed using the universal primers 515F (5′-GTGC CAGCMGCCGCGGTAA-3′) and 806R (5′-GGACTACVSG GGTATCTAAT-3′) with a 12-bp barcode at the 5′ end of each primer (Lauber et al., 2009). Gene library preparation and the PCR reactions followed a previously reported method (Souza et al., 2021). The amplified PCR product were quantified and mixed at equal molar for gel purification. Sequencing was performed on the Illumina NovaSeq PE250 platform according to the protocol of the manufacturer. All sequences were submitted to the NCBI SRA (PRJNA750233).

### Bioinformatics Analysis

Analysis of 16S *rRNA* sequences was performed as previously described (Ding et al., 2019; Chen et al., 2021). High-quality sequences without a technical region (barcode or primers) were used for the following analysis. Chimera sequences were removed jointly by a local BLASTN analysis using the SILVA database (version 138) and ChimeraSlayer analysis. Operational taxonomic unit (OTU) assignment and classification of representative sequences for each OTU were performed using the vsearch software (VSEARCH: a versatile open-source tool for metagenomics) (Rognes et al., 2016) and RDP Classifier (version 2.13) (Wang et al., 2007), respectively. OTUs affiliated to chloroplasts were removed. Statistical comparison, multiple comparison, and plotting were performed using the R software (version 3.6) with different add-On packages. The alpha-diversity index (Chao1 and Pielous’ evenness) was calculated by resampling, for 100 times, of an equal number of reads from each sample. This method helped reduce the biases caused by different read numbers. The unweighted pair group method with arithmetic mean (UPGMA) cluster, based on Bray–Curtis distance, was used to analyze the bacterial beta-diversity. Variations in the bacterial community explained by different parts of the root or soil were analyzed using the R add-On package “Vegan” (Zhang et al., 2016). Discriminative taxa were identified by multiple comparisons using a negative binomial model (Hothorn et al., 2008). Bacterial genera that were positively correlated with each other (Spearman’s rank coefficient > 0.6 and *p* < 0.01) were subjected to co-occurrence network analysis using the Gephi software (version 0.91). *In vitro* antagonists against *Phytophthora capsici* isolated from a previous study using the same soil (Li et al., 2019) were used to estimate their spatial distribution pattern in the rhizosphere. Subsequences of the 16S *rRNA* gene between 515F and 806R of each antagonist were extracted, and the unique phylotype was extracted using the software Vsearch. These unique phylotypes were mapped against the 16S *rRNA* sequence library with a minimum identity of 99% using a standalone BLASTN analysis. All tools mentioned above were implemented in a galaxy instance^1^.

## Results

### Spatial Variation in the Rhizospheric Bacterial Community Was Greater Than the Effect of Different Soils

**FIGURE 1:**
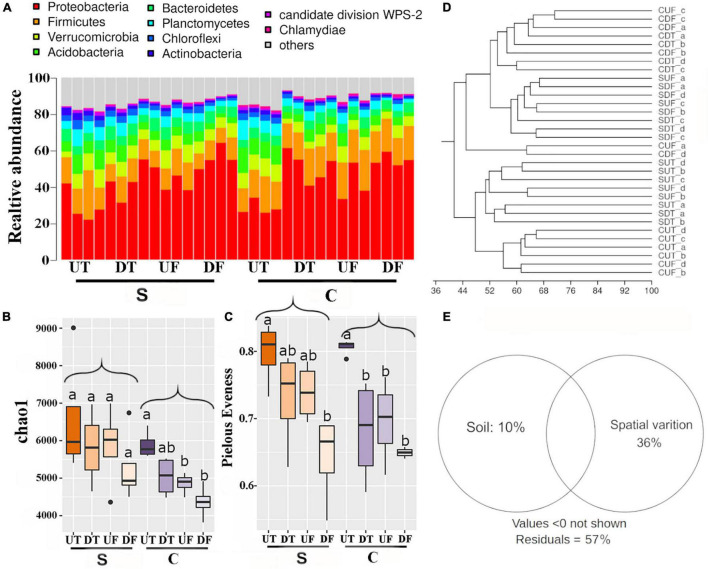
Bacterial diversity in the rhizosphere of the upper (U) or lower (D) part of the taproot (T) or fibrous (F) roots treated by the disease-suppressive (S) or non-suppressive soil (C). Relative abundance of dominant phyla **(A)**, Chao1 richness **(B)**, Pielous’ evenness **(C)**, UPGMA cluster **(D)**, and variation explained by different soils or different parts of roots **(E)**. Significant differences in bacterial alpha-diversity among four parts of root under disease-suppressive or non-suppressive soil are indicated by different letters.

Bacterial communities in different parts of the root systems were analyzed by Illumina sequencing of the 16S *rRNA* gene fragments. A total of 13,404,282 sequences were acquired and 146,369 sequences affiliated with chloroplasts were removed for further analysis. The remaining sequences were grouped into 69,622 OTUs. The most abundant phyla were Proteobacteria (44.8%), Firmicutes (15.3%), Verrucomicrobia (6.8%), Acidobacteria (6.2%), Bacteroidetes (5.3%), and Planctomycetes (4.9%) (Figure 1A). The relative abundance of Proteobacteria was lower in the upper taproot than in the other parts of the root for both soil types (Figure 1A and Supplementary Table 1). For both soil types, bacterial richness and evenness in the rhizosphere decreased from the upper taproot to the upper fibrous root, the lower taproot, and the lower fibrous root (Figures 1B,C). A significant difference between the upper taproot and the lower fibrous root was observed for bacterial evenness in both soil treatments and for richness only in the non-suppressive soils (Figures 1B,C). The bacterial richness was significantly (*p* < 0.05) higher in plants treated with the diseases-suppressive soil than in those treated with the non-suppressive soil (Figure 1B). The bacterial evenness was comparable between the suppressive and non-suppressive soils (Figures 1B,C and Supplementary Table 2). UPGMA cluster analysis indicated that bacterial communities mainly differed between the upper and lower parts of roots (Figure 1D). Variation partition analysis revealed that spatial variation (38.0%) was much greater than that explained by different soils (9.0%) (Figure 1E).

### Discriminative Taxa Between Different Parts of Roots in the Two Soils

**FIGURE 2:**
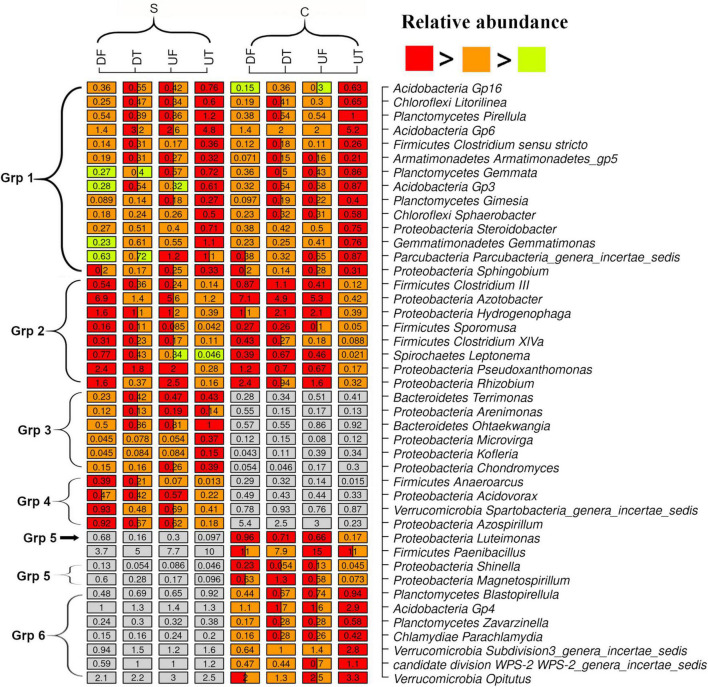
Genera with significantly different relative abundance in the rhizosphere of the upper (U) or lower (L) part of the taproot (T) or fibrous (F) roots treated by the disease-suppressive (S) or non-suppressive soil (C). Significant difference is indicated by a different color. A box with two colors indicates no significant difference from other treatment containing one of the two colors.

Multiple comparisons were performed to identify the dominant (relative abundance of one sample > 0.5%) taxa with contrasting spatial distribution in the rhizosphere of pepper (Figure 2). Most discriminative genera could be assigned to six groups according to their response patterns (Figure 2). The genera in group 1 were commonly enriched in the upper taproot of the plant, and the majority were affiliated with Acidobacteria (e.g., *Gp16*, *Gp3*, and *Gp6*), Planctomycetes (e.g., *Pirellula*, *Gemmata*, and *Gimesia*), Chloroflexi (e.g., *Litorilinea* and *Sphaerobacter*), and Proteobacteria (e.g., *Steroidobacter* and *Sphingobium*) (Figure 2). In addition, few genera, such as *Clostridium sensu stricto*, *Armatimonadetes gp5*, *Gemmatimonas*, and *Parcubacteria_genera_incertae_sedis*, which included members were also commonly enriched in the taproots (Figure 2). Genera (e.g., *Azotobacter*, *Hydrogenophaga*, *Pseudoxanthomonas*, *Rhizobium*, *Clostridium IlI*, *Sporomusa*, *Clostridium XIVa*, and *Leptonema*) in the group 2 commonly decreased in the taproots (Figure 2). The other four groups consisted of genera specifically enriched (groups 3 and 6) or decreased (groups 4 and 5) in the taproot treated with disease-suppressive or non-suppressive soil (Figure 2). Among them, relatively abundant genera such as *Anaeroarcus*, *Acidovorax*, and *Magnetospirillum*, which are known to contain anaerobes, were also lowest in the taproot of pepper treated with disease-suppressive or non-suppressive soil (Figure 2).

### Co-occurrence Network

**FIGURE 3:**
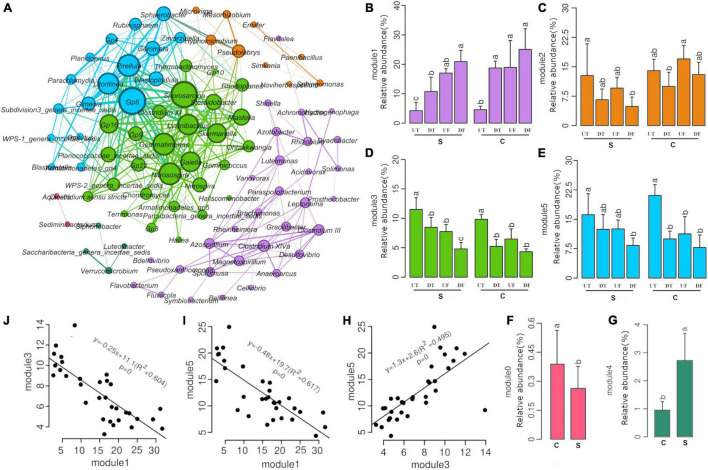
Co-occurrence network analysis of dominant bacteria **(A)**, relative abundance of modules **(B–G)**, and significantly correlated modules **(H–J)**.

A total of 91 genera that were positively correlated with each other were subjected to co-occurrence network analysis (Figure 3). The majority of them were affiliated with Proteobacteria (32 genera), Firmicutes (14 genera), Bacteroidetes (10 genera), Acidobacteria (8 genera), Planctomycetes (7 genera), and Verrucomicrobia (5 genera). These co-occurring genera formed six hubs, including two enriched hubs (modules 3 and 5 in green and cyan, respectively) and one decreased hub (module 1 in purple), in the taproot of pepper (Figure 3A). Correlation analysis further revealed that modules 3 and 5 were positively correlated with each other (Figure 3H), and both were negatively correlated with module 1 (Figures 3I,J). Interestingly, all co-occurring genera affiliated with Acidobacteria or Planctomycetes were among module 3 or 5 (Figure 3A), suggesting that these taxa might prefer the microbial niche in the taproot of pepper. In addition, module 3 also contained nitrifying bacterial taxa, such as *Nitrosospira* and *Nitrospira* (Figure 3A). Module 1 consisted of several bacterial genera adapted to anaerobic and anoxic environments, such as *Clostridium III* or *Clostridium XIVa*, *Magnetospirillum*, and *Anaeroarcus* (Figure 3A). No clear spatial distribution pattern was observed for modules 0 and 4 (Figure 3A). Module 4 was significantly enriched in the treatment with disease-suppressive soil in contrast to module 0 (Figures 3F,G).

### Beneficial Bacteria Tend to Be Enriched in the Upper Taproots

**FIGURE 4:**
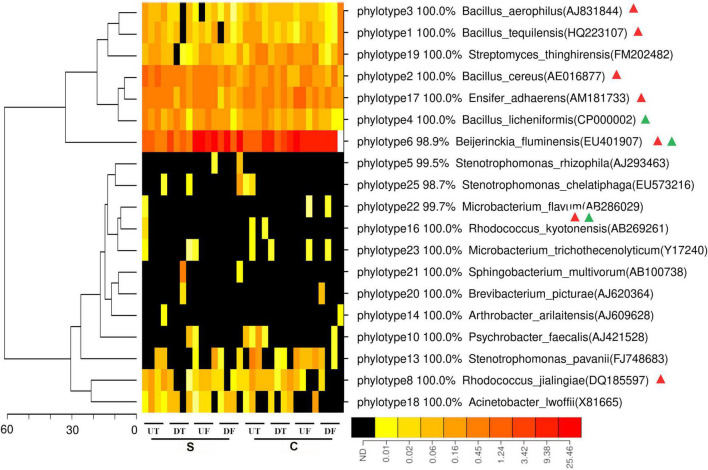
Heatmap analysis of *in vitro* antagonist belonging to each phylotype in the rhizosphere of the upper (U) or lower (D) part of the taproot (T) or fibrous (F) roots treated by the disease-suppressive (S) or non-suppressive soil (C). Those phylotypes with significantly different relative abundance at four parts of roots are indicated by green or red triangle for the disease-suppressive or non-suppressive soil, respectively. The minus symbol indicates decreased relative abundance in the suppressive soil. Phylotype that significantly differed at relative abundance in the rhizosphere of different root parts under suppressive or non-suppressive soil is indicated by red or green triangle, respectively.

To estimate the spatial distribution of *in vitro* antagonists against *P. capsici* along the rhizosphere of pepper, 24 unique phylotypes acquired from a previous study using the same soil microbiome were mapped against the 16S *rRNA* gene fragment. A total of 19 phylotypes could be mapped to 132,713 sequences, accounting for 1.0% of the 16S *rRNA* gene fragments acquired. Interestingly, the percentages of mapped sequences varied between the different parts of the roots (Figure 4). Only 0.37 and 0.43% of sequences could be mapped for the upper taproot of pepper treated by the disease-suppressive or non-suppressive soil, respectively, in contrast to 1.7% for the fibrous roots (Supplementary Figure 1). Among them, the phylotypes similar to *Beijerinckia fluminensis* were most abundant and its relative abundance tended to be higher in the taproot than in the fibrous roots (Figure 4). Seven phylotypes were distributed distinctly along the rhizosphere of pepper treated by the disease-suppressive soil, in contrast to the two phylotypes in the non-suppressive soil (Figure 4). Four out of these seven phylotypes were similar to *Beijerinckia tquilensis*, *Beijerinckia aerophilus*, *Beijerinckia cereus*, and *Beijerinckia firmus*, and their relative abundance was highest in the taproot of pepper (Figure 4). In the treatment with the non-suppressive soil, the phylotype similar to *Beijerinckia licheniformis* was also more abundant in the taproot of pepper (Figure 4).

## Discussion

### Distinct Bacterial Communities Along the Rhizosphere of Pepper

Previously, the rhizospheric microbiome has been frequently studied by using the whole system, in which the heterogeneity in the rhizosphere may be neglected. Herein, a distinct spatial distribution of the bacterial community along the rhizosphere of pepper was detected, indicating that niche differentiation within the rhizosphere might also be considerable. Bacterial alpha-diversity was frequently lower in the rhizosphere than in the corresponding bulk soil, possibly because the plant root only recruits a fraction of soil microorganisms into its rhizosphere (Fierer, 2017; Zhou et al., 2020). Here, the lowest bacterial richness and evenness were detected at the lower fibrous roots in both treatments, suggesting that the selective pressure exerted by plant roots might be strongest at the lower fibrous root of pepper. Root exudates are the major driving forces of microbial communities in the rhizosphere (Berg and Smalla, 2009; Turner et al., 2013). Root hair or fibrous roots often release large quantities of metabolites into the soil (Pandey et al., 2021). Interestingly, the bacterial alpha-diversity in the upper part of fibrous root tends to be higher than in the lower part. Compared with the lower parts, oxygen might be more easily dissolved in the upper parts, which may favor the decomposition of metabolites released by fibrous roots. A slight increase in bacterial richness in the rhizosphere of pepper treated by the disease-suppressive soil agrees with the findings of a previous study (Li et al., 2019). However, it is still premature to state that high bacterial diversity in the rhizosphere may lead to increased resistance to plant diseases, as several other physicochemical properties may influence the assemblage of microbiome in the rhizosphere (Schreiter et al., 2014; Wahdan et al., 2021).

The spatial variation of bacterial community composition along the rhizosphere of pepper was much greater than that explained by different soils, suggesting that the properties of microbial niches in the rhizosphere might be largely determined by the type of root (fibrous root or taproot) and the surroundings at the microscale. This finding agrees with the fact that microbial scale heterogeneity in the soil/rhizosphere, which was largely disregarded, is important for understanding the microbial ecology in soil (Nunan, 2017). Additionally, several biotic/abiotic factors, such as nutrients and bulk density, which could shift the microbial community in the rhizosphere, were also able to change the topology of plant roots (Glimskär, 2000; Wang et al., 2015; Tang et al., 2020). Thus, it is possible that some changes in microbial communities in the rhizosphere were attributed to alterations in the root system in the soil. Additionally, bacterial communities in the rhizosphere of pepper were significantly different between the disease-suppressive and non-suppressive soils, which is in agreement with previous studies. Our results suggest that such differences might be also vary spatially along the rhizosphere and that the upper taproot might be most influenced by the soil. Taken together, all these findings indicate that different roots and soil microbiomes might synergistically shape the spatial distribution of microbiomes in the rhizosphere of pepper.

### Spatial Distribution of Bacterial Taxa Along the Rhizosphere of Pepper and the Implication on Microbial Ecology

Acidobacteria are abundant in soils and represent a significant fraction of the soil bacterial community. Previously, Acidobacteria were largely regarded as oligotrophy (Fierer et al., 2007; Kielak et al., 2016), which may not outcompete in the rhizosphere with excessive nutrients. Here, acidobacterial subgroups 3, 4, 6, and 16 were more abundant in the rhizosphere of the upper taproot. The prevalence of acidobacterial subgroups was also detected in the rhizosphere of several other crops, such as potatoes and leek (Rocha et al., 2013), soybean (Navarrete et al., 2013), and tea (Wang M. et al., 2021), suggesting that some Acidobacteria might also be competent in utilizing root depositions. So far, only dozens of Acidobacteria have been cultivated, which limits our understanding of their physiology (Kalam et al., 2020). However, physiological and genomic studies on cultivable acidobacterial have indicated that some Acidobacteria can degrade xylan (Kielak et al., 2016), which is a major polysaccharide in primary cell walls. However, it remains to be elucidated whether the enrichment of acidobacterial subgroups in the rhizosphere at the upper taproot is associated with their ability to degrade polysaccharides. Interestingly, the module enriched at the taproot also contains *Nitrosospira* and *Nitrospira*, members that have the ability to live an aerobic chemoautotrophic lifestyle by ammonia oxidation (Norton et al., 2008; Lagostina et al., 2015; Kuypers et al., 2018). The relative abundance of anaerobic genera such as *Clostridium IlI*, *Sporomusa*, and *Clostridium XIVa* was enriched in the lower taproot or fibrous roots, and these taxa were often prevalent in anaerobic environments with abundant organic materials (Nevin et al., 2011; Edwards et al., 2013; Ðapa et al., 2013). Genera such as *Rhizobium*, *Azotobacter*, and *Azospirillum* were enriched at the lower fibrous roots, which is in agreement with the fact that nitrogen fixation occurs only under anaerobic conditions (Hayat et al., 2010; Meena et al., 2017; Huang et al., 2021). Other genera such as *Hydrogenophaga* and *Magnetospirillum* are also known to adapt to anaerobic conditions by nitrate respiration (Golby et al., 2012). In summary, these findings indicate that the availability of oxygen may also play an important role in the spatial distribution of different taxa in the rhizosphere of pepper, in addition to root exudates.

### Preferential Colonization of *Bacillus* Antagonists in the Upper Taproot of Pepper

Competition for microbial niches has been proposed as a mechanism employed by beneficial microorganisms to fight against phytopathogens (Götz et al., 2006). Previously, *Bacillus* antagonists in the disease-suppressive soil used in this study may contribute to the suppression of pepper light caused by *P. capsici* (Li et al., 2019). *In silicon* analysis revealed that four isolated *Bacillus* antagonists preferentially colonized the taproot of pepper treated with disease-suppressive soils. This result indicated that the upper taproot might be a hot spot where the *Bacillus* antagonists interacted with the phytopathogen *P. capsici.* This finding agrees with several other studies based on green fluorescence protein-labeled antagonists, in which many antagonists, including *Bacillus*, were preferentially colonized at the lateral root junctions (Liu et al., 2006).

It is also worth noting that the spatial distribution of the bacterial community was studied by dividing the roots systems into four rough compartments, and the microbial communities at finer scale have not been resolved. In addition, the physicochemical conditions of the root pouch differed from those in the soil. Thus, further analysis of the microbial community at a finer scale may provide more details on the spatial distribution of the bacterial community in the rhizosphere. In conclusion, distinct spatial distribution of bacterial community in the rhizosphere of pepper with largely aerobic heterotrophic (including *Bacillus* antagonists against *P. capsici*) and chemotrophic taxa at the upper root and anaerobic taxa (including heterotrophic or diazotrophic, nitrate respiration) at the lower taproot or fibrous roots was found, thus highlighting the importance of root exudates and the availability of oxygen for the reassembling of the rhizosphere microbiome.

## Data Availability Statement

The datasets presented in this study can be found in online repositories. The names of the repository/repositories and accession number(s) can be found below: https://www.ncbi.nlm.nih.gov/, PRJNA750233.

## Author Contributions

G-CD, HL, NW, JL, and YW designed the experiments, wrote the manuscript, and analyzed the data. HL, JD, YL, and XD performed the experiments. All authors participated in the survey, sample collection of organic greenhouse agriculture in different regions, and reviewed the manuscript.

## Conflict of Interest

The authors declare that the research was conducted in the absence of any commercial or financial relationships that could be construed as a potential conflict of interest.

## Publisher’s Note

All claims expressed in this article are solely those of the authors and do not necessarily represent those of their affiliated organizations, or those of the publisher, the editors and the reviewers. Any product that may be evaluated in this article, or claim that may be made by its manufacturer, is not guaranteed or endorsed by the publisher.

## Footnotes

1
www.freebioinfo.org

## Supplementary Material

Supplementary Figure 1 Average relative abundance of reads mapped against all *in vitro* antagonists. Significant differences (*p* < 0.05, multiple comparisons under negative binomial model) are indicated by different letters.
