# Supplementary figures and images for: Spatial Distribution of the Pepper Blight (Phytophthora capsici) Suppressive Microbiome in the Rhizosphere
Source: Front Plant Sci. 2022 Jan 21;12:748542. doi: 10.3389/fpls.2021.748542 (PMC8813743; doi:10.3389/fpls.2021.748542)

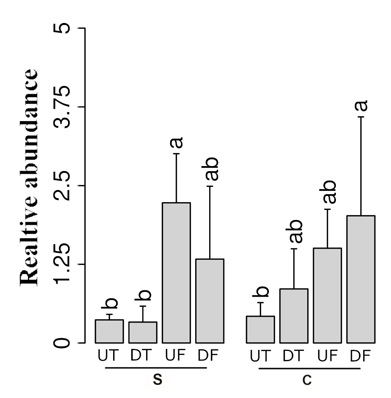

Supplement: Supplementary Figure 1 — Average relative abundance of reads mapped against all in vitro antagonists. Significant differences (p < 0.05, multiple comparisons under negative binomial model) are indicated by different letters. [file Image_1.TIFF]
