# Supplementary material for: Spatial Distribution of the Pepper Blight (Phytophthora capsici) Suppressive Microbiome in the Rhizosphere
Source: Front Plant Sci. 2022 Jan 21;12:748542. doi: 10.3389/fpls.2021.748542 (PMC8813743; doi:10.3389/fpls.2021.748542)
Supplement: Supplementary file 2 [file Table_1.DOCX]

Table S1. Relative abundance of dominant phyla

| Phylum | S_UT_a | S_UT_b | S_UT_c | S_UT_d | S_DT_a | S_DT_b | S_DT_c | S_DT_d | S_UF_a | S_UF_b | S_UF_c | S_UF_d | S_DF_a | S_DF_b | S_DF_c | S_DF_d | C_UT_a | C_UT_b | C_UT_c | C_UT_d | C_DT_a | C_DT_b | C_DT_c | C_DT_d | C_UF_a | C_UF_b | C_UF_c | C_UF_d | C_DF_a | C_DF_b | C_DF_c | C_DF_d |
| --- | --- | --- | --- | --- | --- | --- | --- | --- | --- | --- | --- | --- | --- | --- | --- | --- | --- | --- | --- | --- | --- | --- | --- | --- | --- | --- | --- | --- | --- | --- | --- | --- |
| Proteobacteria | 42.21 | 25.46 | 22.21 | 27.77 | 43.32 | 31.61 | 43.02 | 55.51 | 51.14 | 38.77 | 46.60 | 38.53 | 50.11 | 55.08 | 64.67 | 55.24 | 26.48 | 34.39 | 26.05 | 27.85 | 61.74 | 55.37 | 40.96 | 45.64 | 54.68 | 33.61 | 53.74 | 38.24 | 53.67 | 59.77 | 52.26 | 55.16 |
| Firmicute | 14.46 | 13.83 | 27.29 | 12.14 | 9.58 | 14.40 | 13.02 | 11.15 | 9.13 | 11.69 | 14.84 | 15.27 | 8.66 | 10.90 | 8.32 | 12.36 | 13.01 | 15.39 | 20.06 | 15.32 | 13.62 | 15.00 | 20.52 | 17.03 | 15.61 | 20.19 | 18.22 | 22.49 | 17.31 | 18.26 | 15.01 | 18.79 |
| Verrucomicrobia | 3.04 | 8.11 | 9.18 | 10.03 | 6.36 | 8.07 | 7.06 | 5.33 | 5.85 | 9.94 | 7.14 | 9.73 | 6.51 | 6.12 | 5.64 | 7.87 | 8.41 | 8.34 | 9.85 | 9.39 | 2.56 | 5.81 | 3.69 | 6.50 | 4.27 | 8.87 | 5.20 | 7.00 | 3.34 | 3.73 | 6.94 | 5.33 |
| Acidobacteria | 5.78 | 10.70 | 6.86 | 10.86 | 7.03 | 9.18 | 5.97 | 4.06 | 5.02 | 6.05 | 4.87 | 6.16 | 5.68 | 3.39 | 2.28 | 3.42 | 14.15 | 9.19 | 9.25 | 10.61 | 3.66 | 4.22 | 6.86 | 6.13 | 4.42 | 7.45 | 3.36 | 4.92 | 5.44 | 1.64 | 4.63 | 2.15 |
| Bacteroidete | 6.88 | 4.67 | 4.48 | 5.21 | 7.54 | 5.18 | 6.18 | 4.06 | 7.27 | 6.84 | 5.15 | 6.62 | 5.39 | 6.65 | 4.01 | 5.68 | 4.24 | 5.74 | 4.87 | 5.39 | 3.41 | 3.17 | 4.88 | 6.17 | 4.50 | 5.06 | 4.27 | 6.15 | 3.44 | 4.83 | 3.47 | 5.48 |
| Planctomycete | 4.28 | 9.25 | 6.56 | 7.28 | 5.21 | 7.23 | 4.46 | 3.60 | 4.20 | 5.89 | 5.10 | 4.60 | 4.57 | 2.86 | 2.83 | 3.44 | 10.82 | 5.78 | 6.72 | 6.64 | 4.15 | 3.20 | 5.12 | 3.71 | 3.64 | 5.98 | 2.75 | 4.07 | 4.64 | 1.22 | 3.71 | 1.88 |
| Chloroflexi | 3.11 | 5.14 | 3.07 | 3.64 | 3.30 | 3.62 | 3.62 | 2.50 | 2.52 | 2.83 | 2.03 | 2.17 | 3.82 | 1.84 | 1.18 | 1.33 | 3.71 | 2.63 | 3.40 | 3.20 | 2.43 | 1.30 | 3.01 | 1.31 | 1.36 | 2.09 | 1.53 | 1.76 | 1.89 | 0.67 | 2.07 | 0.65 |
| Actinobacteria | 4.29 | 3.92 | 2.76 | 2.67 | 2.10 | 1.99 | 1.84 | 1.33 | 1.27 | 1.84 | 1.41 | 1.66 | 1.12 | 1.22 | 0.56 | 1.16 | 1.92 | 2.37 | 2.45 | 2.02 | 1.44 | 0.95 | 1.98 | 1.17 | 1.15 | 1.58 | 1.15 | 1.89 | 1.35 | 1.22 | 1.00 | 1.23 |
| candidate divission WPDSS_-2 | 0.53 | 1.42 | 1.06 | 1.77 | 0.78 | 1.71 | 0.90 | 0.70 | 0.67 | 1.06 | 0.85 | 1.40 | 0.65 | 0.50 | 0.61 | 0.61 | 1.70 | 0.76 | 0.77 | 1.03 | 0.26 | 0.47 | 0.47 | 0.56 | 0.67 | 1.02 | 0.59 | 0.52 | 0.52 | 0.29 | 0.66 | 0.40 |
| Chlamydiae | 0.30 | 0.43 | 0.48 | 0.66 | 0.74 | 0.54 | 0.31 | 0.80 | 0.37 | 0.73 | 0.61 | 0.75 | 0.73 | 0.52 | 0.35 | 0.43 | 1.11 | 1.32 | 1.46 | 1.16 | 0.36 | 0.98 | 1.18 | 1.17 | 0.57 | 1.46 | 1.14 | 1.04 | 0.55 | 0.65 | 1.84 | 0.55 |
