# Supplementary material for: Spatial Distribution of the Pepper Blight (Phytophthora capsici) Suppressive Microbiome in the Rhizosphere
Source: Front Plant Sci. 2022 Jan 21;12:748542. doi: 10.3389/fpls.2021.748542 (PMC8813743; doi:10.3389/fpls.2021.748542)
Supplement: Supplementary file 3 [file Table_2.DOCX]

Table S2 Comparison of bacterial alpha diversity among different parts of roots under disease suppressive or non-suppressive soil treatment.

| Alpha_diversity | S_DF | S_DT | S_UF | S_UT | C_DF | C_DT | C_UF | C_UT |
| --- | --- | --- | --- | --- | --- | --- | --- | --- |
| Chao1 | 5278±996a | 5812±1003a | 5850±1093a | 6588±1647a | 4367±447b | 5038±531ab | 4861±274b | 5885±367a |
| Pielous Eveness | 0.642±0.0663b | 0.731±0.0743ab | 0.739±0.0429ab | 0.798±0.0469a | 0.649±0.00745b | 0.681±0.07725b | 0.696±0.06288b | 0.806±0.01176a |

Note: significant difference was indicated by different letters.
